# Antimicrobial Volatiles of the Insect Pathogen *Metarhizium brunneum*

**DOI:** 10.3390/jof8040326

**Published:** 2022-03-22

**Authors:** Esam Hamid Hummadi, Yarkin Cetin, Merve Demirbek, Nadeems M. Kardar, Shazia Khan, Christopher J. Coates, Daniel C. Eastwood, Ed Dudley, Thierry Maffeis, Joel Loveridge, Tariq M. Butt

**Affiliations:** 1 Faculty of Science and Engineering, Swansea University, Singleton Park, Swansea SA2 8PP, Wales, UK; yarkinaybars.cetin@urv.cat (Y.C.); mervgozde@gmail.com (M.D.); m.kardar@midlothian.education (N.M.K.); shazia.khan@ed.ac.uk (S.K.); c.j.coates@swansea.ac.uk (C.J.C.); d.c.eastwood@swansea.ac.uk (D.C.E.); t.g.g.maffeis@swansea.ac.uk (T.M.); e.j.loveridge@swansea.ac.uk (J.L.); 2 Department of Biotechnology, College of Science, University of Diyala, Baqubah City 32001, Iraq; 3 Faculty of Medicine, Health and Life Science, Swansea University, Singleton Park, Swansea SA2 8PP, Wales, UK; e.dudley@swansea.ac.uk

**Keywords:** *Metarhizium brunneum*, entomopathogenic fungi, antimicrobial compounds, volatile organic compounds, plant pathogens

## Abstract

Fungal volatile organic compounds (VOCs) represent promising candidates for biopesticide fumigants to control crop pests and pathogens. Herein, VOCs produced using three strains of the entomopathogenic fungus *Metarhizium brunneum* were identified via GC-MS and screened for antimicrobial activity. The VOC profiles varied with fungal strain, development state (mycelium, spores) and culture conditions. Selected VOCs were screened against a range of rhizosphere and non-rhizosphere microbes, including three Gram-negative bacteria (*Escherichia coli*, *Pantoea agglomerans*, *Pseudomonas aeruginosa*), five Gram-positive bacteria (*Micrococcus luteus*, *Staphylococcus aureus*, *Bacillus subtilis*, *B. megaterium*, *B. thuringiensis*), two yeasts (*Candida albicans*, *Candida glabrata*) and three plant pathogenic fungi (*Pythium ultimum*, *Botrytis cinerea*, *Fusarium graminearum*). Microbes differed in their sensitivity to the test compounds, with 1-octen-3-ol and isovaleric acid showing broad-spectrum antimicrobial activity. Yeasts and bacteria were inhibited by the same VOCs. Cryo-SEM showed that both yeasts and bacteria underwent some form of “autolysis”, where all components of the cell, including the cell wall, disintegrated with little evidence of their presence in the clear, inhibition zone. The oomycete (*P. ultimum*) and ascomycete fungi (*F. graminearum*, *B. cinerea*) were sensitive to a wider range of VOCs than the bacteria, suggesting that eukaryotic microbes are the main competitors to *M. brunneum* in the rhizosphere. The ability to alter the VOC profile in response to nutritional cues may assist *M. brunneum* to survive among the roots of a wide range of plant species. Our VOC studies provided new insights as to how *M. brunneum* may protect plants from pathogenic microbes and correspondingly promote healthy growth.

## 1. Introduction

The rhizosphere is a highly competitive environment in which microbes exhibit different strategies for survival. Entomopathogenic fungi (EPF) are commonly found in the rhizosphere, using root exudates as a source of nutrition but switching to insects when the opportunity arises [1,2,3,4,5]. EPF form symbiotic relationships with plants, leading to improved plant growth and productivity [1,6]. Plants treated with EPF species, such as *M. brunneum* and *Beauveria bassiana*, usually have more extensive root systems (lateral roots, root hairs), greater biomass (roots, shoots) and higher crop yields than untreated plants [7,8,9]. Besides stimulating plant growth and increasing nitrogen uptake, EPF antagonise plant pathogenic fungi, stimulate plant defences and increase plant tolerance to stress [6,10,11]. Exactly how EPF compete with antagonistic microbes in the rhizosphere is unclear. 

Most rhizosphere microbes produce antimicrobial compounds, which include secretory products, as well as low-molecular-weight volatile organic compounds (VOCs), which differ in their specificity and mode of action [12,13,14,15]. VOCs were also reported to promote or inhibit plant growth, trigger plant resistance and attract other micro- and macro-organisms [14,16,17,18,19,20,21]. Although EPF are known to produce VOCs, very few have been identified and characterised. Some of the VOCs have semiochemical properties while others are toxic to invertebrates, including crop pest species like plant-parasitic nematodes and molluscs [22,23,24,25,26,27]. VOCs allow Collembola to locate *Metarhizium anisopliae* conidia to feed on [28,29] and ants and termites to discriminate between pathogenic and non-pathogenic strains of *M. anisopliae* [30,31]. The current study presented the first evidence that *Metarhizium* VOCs have antimicrobial properties that inhibit a wide range of soil microbes. The significance of these compounds to *Metarhizium* survival in the rhizosphere and protection of plants against plant pathogenic microbes is discussed. An understanding of EPF survival strategies in the rhizosphere could help to not only improve plant productivity but also make more efficacious use of these natural biopesticides in pest management programmes.

## 2. Materials and Methods 

### 2.1. M. brunneum Isolates and Culture Conditions for Collecting VOCs

VOCs of *M. brunneum* isolates ARSEF 4556 (origin: *Boophilus* sp., USA), ARSEF 3297 (origin: *Boophilus* sp., Mexico) and V275 (origin: *Cydia pomonella*, Austria) were collected from cultures produced on four different media known to influence their physiology and virulence [32]. Media included (*w*/*v*): (1) osmotic stress medium (OSM, 8% glucose, 2% peptone, 5.5% agar, 5.5% KCl), (2) high C:N (75:1) medium (9.1% glucose, 1% peptone, 2% agar), (3) intermediate C:N (35:1) medium (4% glucose, 1% peptone, 2% agar) and (4) low C:N (10:1) medium (0.6% glucose, 1% peptone, 2% agar). VOCs were collected 7 and 14 days post-inoculation, corresponding with the mycelial and sporulating stages of the fungi. All media were obtained from Sigma (Poole, UK), except peptone, which was obtained from Oxoid, Hampshire, UK. Cultures were produced using 10 mL medium in 25 mL glass vials incubated in the dark at 25 °C. There were three technical replicates per treatment (isolate, culture medium) with the whole experiment being repeated on two independent occasions.

### 2.2. Collection and Identification M. brunneum VOCs

Headspace VOCs were collected from the above treatments using a 50/30 mm divinylbenzene/carboxen/polydimethylsiloxane (DVB/CAR/PDMS) SPME fibre (Supelco, Bellefonte, PA, USA) and analysed using an Agilent 6890N Gas Chromatograph equipped with an HP-5MS fused capillary column (30 m × 0.25 mm × 0.25 µm film thickness), which was interfaced directly with an Agilent 5975 mass spectrometer. Helium was used as the carrier gas with a constant flow of 1.0 mL/min. Immediately after collecting the VOCs, the SPME needle was inserted manually into the injection port (230 °C; splitless mode) of the GC-MS for thermal desorption and held for 2 min. After desorption, the oven was held at 40 °C for 2 min, then the temperature was raised to 200 °C at a rate of 3 °C/min. Finally, the temperature was raised to 270 °C at a rate of 8 °C/min and held at 270 °C for 10 min. Mass spectra were scanned repeatedly over 35–650 amu. Ionisation was performed in electron impact (EI) mode at 70 eV. A blank run was performed after each analysis to confirm that no residual compound was polluting the fibre or column. Total ion current (TIC) chromatograms were integrated without any correction for co-elution and results were expressed as the percent of the total peak area. All peaks were identified from their mass spectra via comparison with spectra in Wiley Registry (9th edition) and NIST11 (National Institute of Standards and Technology, Gaithersburg, MD, USA) libraries. Identifications were confirmed by comparing the retention time, molecular ions and fragmentation pattern with authentic standard samples. The peaks observed in the control (blank media without fungus) were excluded from the samples during the sample analysis. All reference compounds used for identification were purchased from Sigma Aldrich (Poole, UK), except methyl 2-ethylhexanoate, which was synthesized by reacting 2-ethylhexanoic acid with methanol in the presence of concentrated sulphuric acid (catalyst), as outlined by [33].

### 2.3. Evaluation of VOC Antimicrobial Activity 

Fourteen *M. brunneum* VOCs, validated using authentic standards (Supplementary Table S1), were tested against a range of rhizosphere and non-rhizosphere microbes. Details of the test organisms, strains, source and ecological relevance are summarised in Supplementary Table S2. The antimicrobial activity of *M. brunneum* VOCs was determined using the disc volatilisation and radial growth assays. Pure compounds from commercial sources were used for these and subsequent studies (Supplementary Table S1).

The disc volatilisation assay was based on the methodology described by [34]. Briefly, test bacteria and *Candida* yeast were grown on Luria Bertani Agar (LBA; pH 7.5) and SDA, respectively. The cultures were incubated at 35 °C for 24 h and fresh inoculum was prepared by diluting the bacteria and yeasts in sterile PBS, pH 7, and adjusted to the 0.5 McFarland standard. Bacterial and yeast cell densities were measured in a spectrophotometer at 500 nm and 530 nm wavelengths, respectively [35,36]. The freshly prepared bacteria and yeasts were inoculated onto LBA and SDA media, respectively. An 8 mm diameter filter paper disc was placed on a coverslip attached to the inside of a Petri dish lid and loaded with 10 µL of the volatile compound before inverting the culture plate over the lid and sealing with two layers of Parafilm. After 24 h of incubation at 35 °C, the diameter of the clear (inhibition) zone was measured in millimetres. Blank discs served as negative controls. To determine whether the microbes could recover, the paper disc was removed and the plates were ventilated for 15 min and incubated for another 24 h at 35 °C. There were four replicates per treatment and the experiment was repeated two times. 

The radial growth assay was adapted from the method described by [37]. The filamentous oomycete (*P. ultimum*) and fungi (*F. graminearum*, *B. cinerea*) were grown on PDA at 27 °C for 7 days. Plugs (8 mm diameter) were removed from the centre of the unseeded PDA using a sterile cork borer and replaced with mycelial plugs of the actively growing test fungus. The inoculated plates were treated with the volatile compounds as described above and incubated at 27 °C with the colony diameters being measured (in millimetres) 5 days post-exposure. For the control, a blank paper disc was used. The percent growth inhibition (%GI) was calculated using the formula: %GI = (R1 − R2)/R1 × 100 where R1 is the radial growth of the control and R2 is the radial growth of the treated oomycete/fungi. The inhibition of these organisms was compared with that of the EPF *Metarhizium anisopliae* ARSEF 5469, obtained from the USDA ARS culture collection. 

### 2.4. Scanning Electron Microscopy (SEM)

The effect of *M. brunneum* VOCs on bacteria and yeast cells was investigated using SEM. A sliver of agar was taken from untreated and treated cultures with particular attention being given to healthy cells and cells in the centre and edge of the inhibition (clear) zone. Specimens were fixed to the Cryo-SEM sample holder with cryogenic glue, then plunged into a liquid nitrogen slurry (−190 °C) for rapid freezing. The specimen was then warmed to −90 °C for 10 min to etch away the surface water before being sputter coated with approximately 5 nm of platinum and examined at −130 °C. Imaging was conducted using a Hitachi S4800 field emission microscope equipped with a Quorum PPT2000 cryogenic stage and preparation chamber. 

### 2.5. Data Handling

The disc volatilization and radial growth assays were expressed as means ± standard deviation (mean ± SD). Statistical analysis was performed using one-way analysis of variance (ANOVA) followed by Tukey’s HSD test to compare between the VOCs activity values against each microbe using SPSS software, Version *22* for Windows (IBM SPSS Inc., Chicago, IL, USA). Values from two doses (10 and 15 µL) were compared statistically using multiple t-tests through GraphPad Prism v.7.0 for Windows (GraphPad Software, USA). A *p*-value < 0.05 was considered statistically significant. 

## 3. Results

### 3.1. VOC Profiles Were Influenced by Strain and Culture Medium

**Figure 1:**
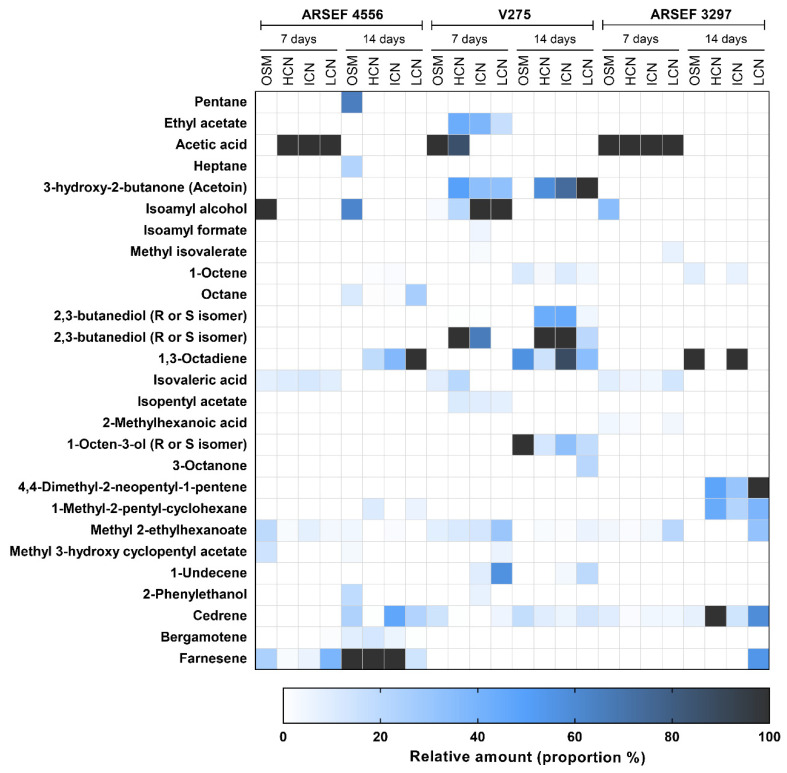
Profiles of volatile organic compounds produced by *M. brunneum* ARSEF4556, V275 and ARSEF3297 on different substrates. The heatmap represents values that were proportional to the compound with the highest peak (100%) in the chromatogram. *n* = 5. OSM, osmotic stress medium; HCN, high C:N medium; ICN, intermediate C:N medium; LCN, low C:N medium. Note: no conidia were present in the ARSEF 4556 at 7 days.

A wide range (>40) of VOCs were produced by *M. brunneum* (ARSEF4556, ARSEF3297, V275), with some compounds being produced more consistently than others (Supplementary Table S3). The relative quantities of each VOC varied between strains, development stage (young mycelial versus older sporulating cultures) and culture conditions (Figure 1). For convenience, the compounds were expressed as a percentage relative to the compound with the largest peak (100%) in the GC-MS chromatogram. VOCs of all three *M. brunneum* strains included a variety of saturated and unsaturated hydrocarbons, alcohols, acids, ketones, esters, terpenes and other assorted compounds (Supplementary Table S3). 

The VOC profiles of 7-day-old non-sporulating cultures differed from the 14-day-old cultures that consisted of mycelium, conidiophores and conidia (Supplementary Table S4). Some compounds were produced by all three strains but not necessarily on all media tested (e.g., acetic acid, isoamyl alcohol, 1-octene, 1,3-octadiene, isovaleric acid, methyl 2-ethylhexanoate, cedrene). Methyl 2-ethylhexanoate and cedrene were the most observed compounds (Figure 1). Acetic acid and isovaleric acid were produced only during the mycelial phase of growth, while pentane, heptane, octane, 1,3-octadiene, 1-octen-3-ol, 3-octanone, 4,4-dimethyl-2-neopentyl-1-pentene, 1-methyl-2-pentylcyclohexane and 4,4,7a-trimethyl-2,4,5,6,7,7a-hexahydro-1H-inden-1-one were only produced during the sporulation phase (Figure 1; Supplementary Table S4). Farnesene was specific to ARSEF 4556 and produced during the mycelial and sporulation phases on all media tested (Figure 1, Supplementary Table S4); it was particularly abundant during the sporulation phase. Cedrene was emitted by V275 and ARSEF 3297 at almost all development stages, but it was only observed in ARSEF 4556 during sporulation. There was a noticeable difference in the class of VOCs produced by each strain, for example, ARSEF 4556 tended to produce alkanes (e.g., pentane, heptane, and octane) and terpenoids (cedrene, bergamotene, farnesene). Strain V275 tended to produce alcohols, esters and ketones, while ARSEF 3297 produced a much smaller range of volatiles. 

*Metarhizium* VOC profiles were influenced by the culture medium (OSM, HCN, ICN and LCN), independent of fungal strain (Figure 1, Supplementary Table S4). Variations in the VOC profiles were evident in OSM, but similar patterns were observed on the other media, albeit in different quantities. In 7-day-old mycelial cultures of *M. brunneum* ARSEF 4556, isoamyl alcohol was more predominant than acetic acid on OSM, but the opposite was observed for cultures grown on HCN, ICN and LCN (Figure 1, Supplementary Table S4). V275 produced fewer VOCs on OSM than on the other media, with the most notable absences being that of ethyl acetate, 3-methyl-2-butanone, 2,3-butanediol and isoamyl acetate. For *M. brunneum* ARSEF 3297, isoamyl alcohol was only produced by 7-day-old cultures grown on OSM. However, 14-day-old cultures produced 4,4-dimethyl-2-neopentyl-1-pentene and 1-methyl-2-pentylcyclohexane on all media except OSM (Figure 1, Supplementary Table S4). 

The 7-day-old non-sporulating cultures of *M. brunneum* differed in the VOC profile from that of the 14-day-old sporulating cultures (Figure 1, Supplementary Table S4). Collectively, the aliphatic C8 compounds, i.e., 1-octene, octane, 1,3-octadiene, 1-octen-3-ol and 3-octanone, were found in older cultures, whereas acetic acid and isoamyl alcohol were exclusively produced by the young non-sporulating cultures. The range of VOCs produced by ARSEF 4556 increased in older sporulating cultures but declined for V275 and remained unaltered for 3297 (Figure 1, Supplementary Table S4). For ARSEF 4556, more alkanes, alkenes and sesquiterpenes were produced, and the abundance of sesquiterpenes increased during sporulation. During the growth phase of V275, C5 derivatives, i.e., isoamyl alcohol, isovaleric acid and isoamyl acetate, were more abundant initially but were replaced by C8 compounds on sporulation. Similarly, in strain 3297, acetic acid, isovaleric acid and 2-methylhexanoic acid were produced during the mycelial phase, while unsaturated hydrocarbons were prominent during sporulation (Figure 1, Supplementary Table S4).

### 3.2. VOC Antibacterial and Antifungal Activity

**Figure 2:**
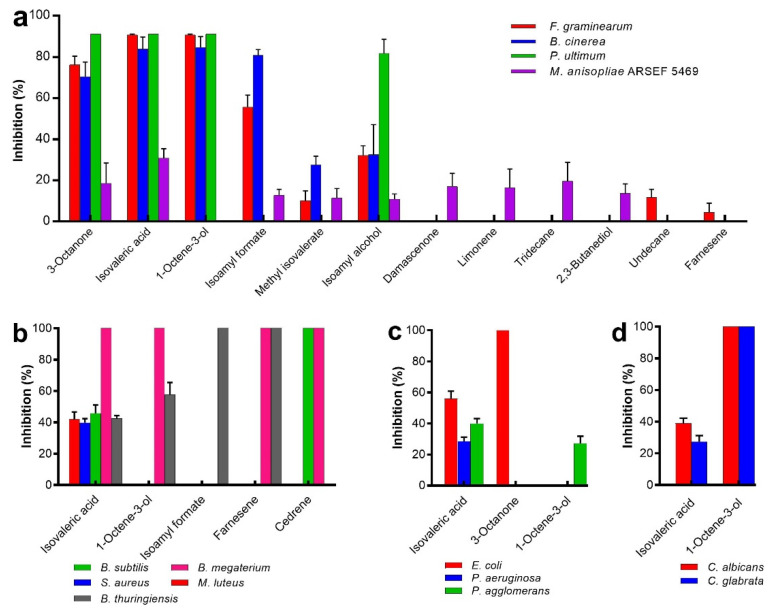
Inhibition (%) of growth of (**a**) filamentous fungi, (**b**) G+ bacteria, (**c**) G− bacteria and (**d**) yeasts when exposed to VOCs. The bacteria and yeasts were examined 24 h post-exposure (to 10 µL VOC) and the filamentous fungi 5 days post-exposure. Values represent mean (±SD) of two experiments, each with 4 technical replicates per treatment.

*Metarhizium anisopliae* ARSEF 5469 radial growth was partially reduced by the 14 test compounds used (Supplementary Table S1), where isovaleric acid and 3-octanone caused the greatest reductions at 34 ± 9.92% and 26 ± 5.04% inhibition, respectively (Figure 2A–D). In contrast, the plant pathogenic fungi/oomycete were far more sensitive, being strongly inhibited (85–100%) by isovaleric acid and 1-octen-3-ol (Figure 2A–D). 

The yeasts, filamentous fungi and oomycete differed in their sensitivity to *M. brunneum* VOCs. The yeasts, i.e., *C. albicans* and *C. glabrata*, were only affected by isovaleric acid and 1-octen-3-ol, with the latter causing 100% inhibition (Figure 2D). The filamentous fungi *F. graminearum* and *B. cinerea* were inhibited by a wider range of VOCs than the other microbes (Figure 2A). *F. graminearum* was sensitive to most (8/14) of the compounds tested, with inhibition ranging from 5 ± 2% (farnesene) to 100 ± 0.00% (isovaleric acid, 1-octen-3-ol). Inhibition of *B. cinerea* ranged from 28 ± 4% (methyl isovalerate) to 85 ± 4.9% (1-octen-3-ol) (Figure 2A). The oomycete, i.e., *P. ultimum*, was highly sensitive to only four compounds, with inhibition ranging from 82 ± 6.36% (isoamyl alcohol) to 100% (3-octanone, isovaleric acid, 1-octen-3-ol) (Figure 2A). 

**Figure 3:**
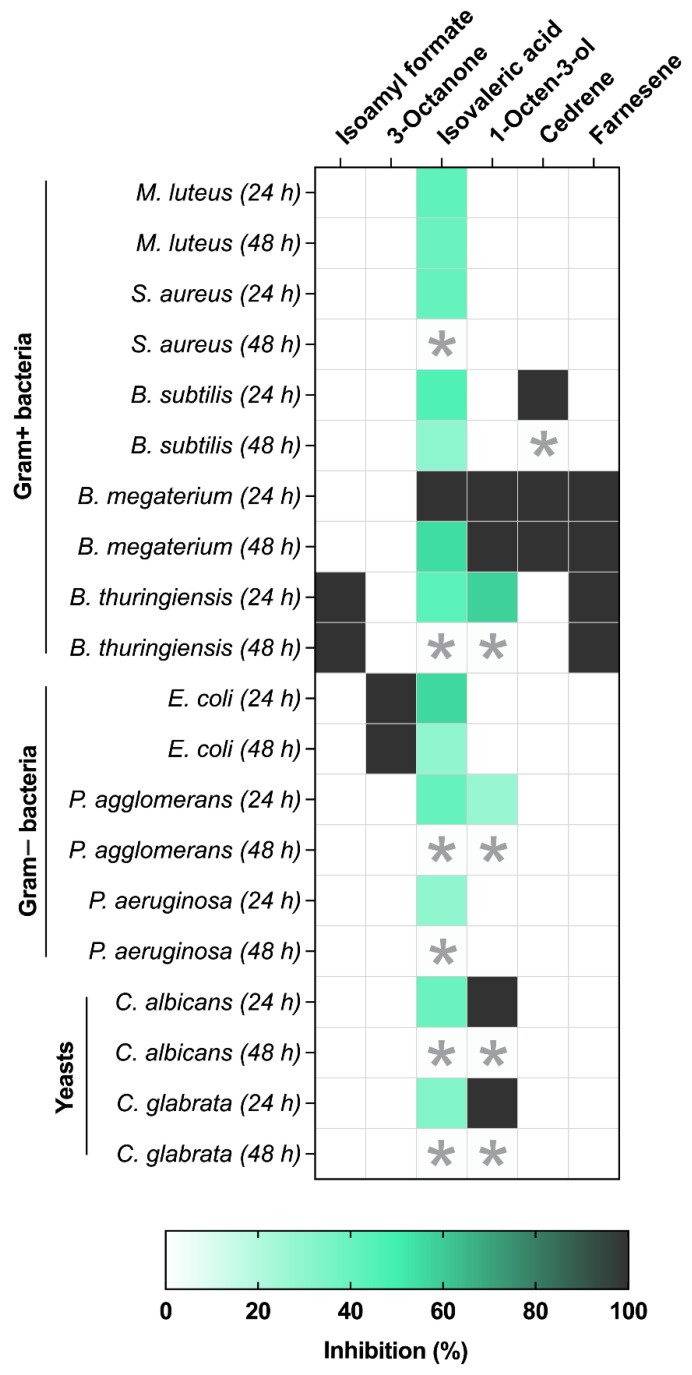
Growth inhibition (%) of bacteria and yeast exposed to volatile organic compounds. Microbial growth was recorded at 24 and 48 h post-exposure to 10 µL VOC. Values represent the mean (±SD) of two independent experiments, consisting of 4 technical replicates per treatment. An asterisk (*) denotes growth recovery.

Only one compound, isovaleric acid, inhibited all test bacteria, with inhibition ranging from 29 ± 2.41% (*P. aeruginosa*) to 100% (*B. megaterium*) after 24 h (Figure 2B,C and Figure 3). Isoamyl formate and farnesene totally inhibited *B. thuringiensis* (Figure 2B and Figure 3). 3-Octanone totally inhibited *E. coli* without affecting any other test bacterium (Figure 2C and Figure 3). Interestingly, *B. megaterium* showed the greatest sensitivity to isovaleric acid, 1-octen-3-ol, cedrene and farnesene, which caused 100% inhibition. Cedrene caused 100% inhibition of *B. subtilis* (Figure 2B and Figure 3).

Since the VOCs are ephemeral, the recovery of the bacteria and yeast was observed 48 h after treatment. The recovery depended on the compound and test organism. Whereas the majority of microbes recovered to various degrees, some failed to do so and were presumed dead. The yeast recovered totally once the VOC source was removed, rapidly colonising the clear (inhibition) zone (Figure 3). Full recovery was also recorded for *P. aeruginosa*, *P. agglomerans*, *B. thuringiensis* and *S. aureus* exposed to isovaleric acid, for *P. agglomerans* and *B. thuringiensis* exposed to 1-octen-3-ol and for *B. subtilis* exposed to cedrene (Figure 3). Partial recovery (55 ± 7.4%) was observed for *B. megaterium* exposed to isovaleric acid. Full inhibition (100%) was recorded for *E. coli* exposed to 3-octanone 48 h post-treatment. Full inhibition was also recorded for *B. thuringiensis* exposed to isoamyl formate and farnesene and for *B. megaterium* exposed to 1-octen-3-ol, cedrene and farnesene (Figure 3).

**Figure 4:**
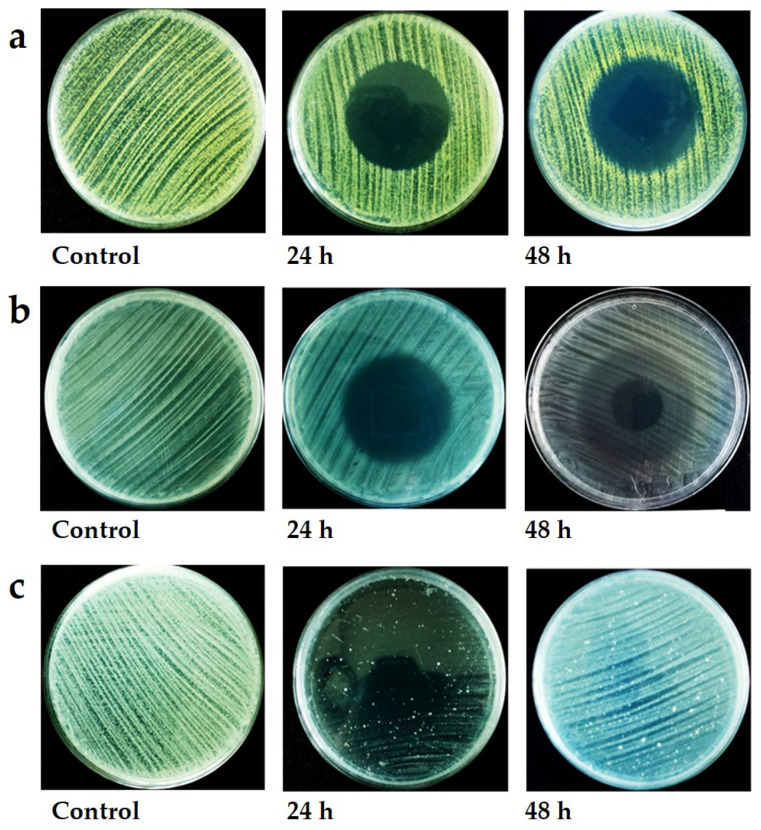
Appearance of bacterial cultures when exposed to selected volatile organic compounds. (**a**) *M. luteus* NCIB 8553 culture exposed to 10 µL isovaleric acid. Note the dense growth at the edge of the inhibition zone. (**b**) *E. coli* NCIB 8277 culture exposed to 10 µL isovaleric acid. Note the dense growth in the uninhibited zone and moderate growth in the recovery zone, with a clear zone at the centre where the concentration of the chemical was the highest. (**c**) *B. subtilis* NCIP 3610 culture exposed to 10 µL cedrene. Note that the culture was mostly inhibited, except for a few colonies when exposed 24 h to this VOC, but total recovery occurred once the culture was ventilated and allowed to incubate for a further 24 h.

Where recovery was observed, bacterial and, to some extent, yeast colonies grew from the edge of the colony (interface between the surviving culture and zone of inhibition) towards the centre of the clear zone (Figure 4A). In some instances, growth at the interface was extremely vigorous, resulting in a pronounced collar (Figure 4A), but in other instances, growth was faint, resulting in a halo effect (Figure 4B). Occasionally, small colonies arose within the clear zone presumably from cells protected by other cells (Figure 4C). 

**Figure 5:**
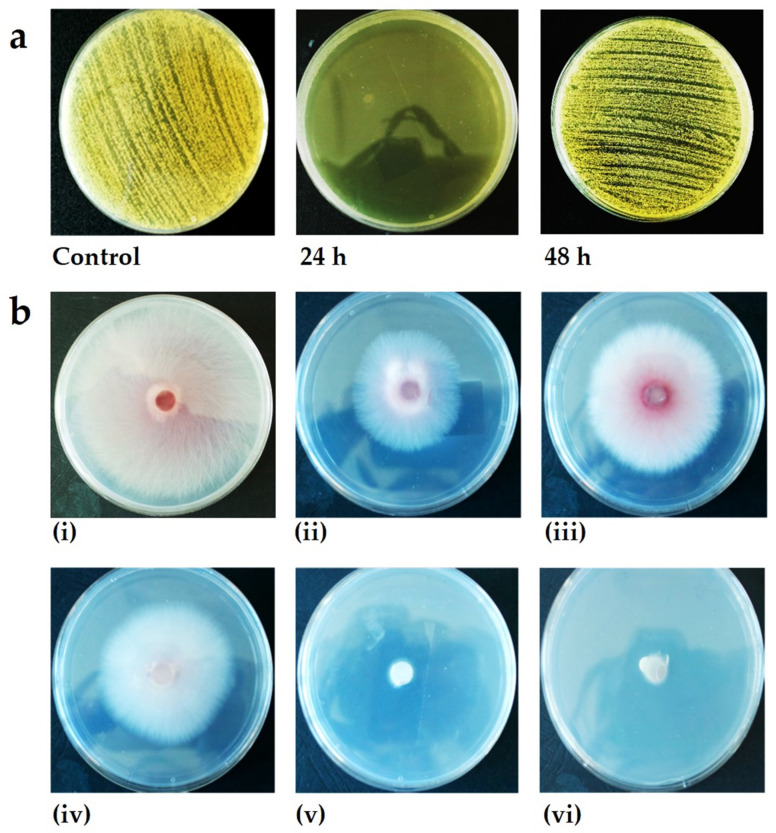
Inhibition of fungal cultures exposed to selected volatile organic compounds. (**a**) *Candida albicans* NCYC3778 exposed to 10 µL 1-octene-3-ol. Substantial inhibition was achieved within 24 h, but the cells appeared to recover after ventilation and a further 24 h incubation (i.e., 48 h). (**b**) Five-day-old cultures of *F. graminearum* in the (**i**) absence and presence (15 µL) of (**ii**) 3-octanone, (**iii**) isoamyl alcohol, (**iv**) isoamyl formate, (**v**) 1-octene-3-ol and (**vi**) isovaleric acid.

**Figure 6:**
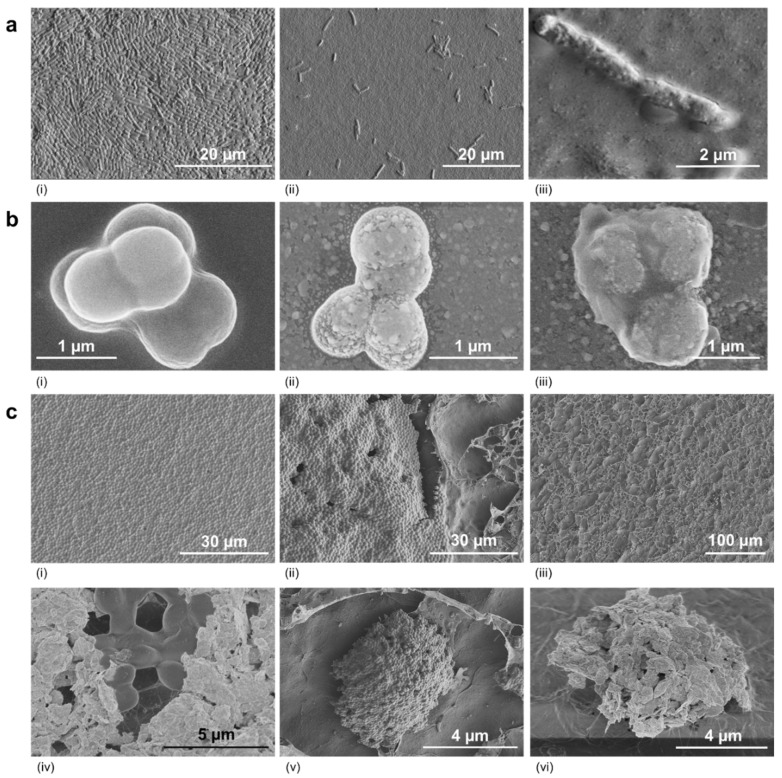
Scanning electron micrographs of bacteria exposed to selected volatile organic compounds. (**a**) *E. coli* in the (**i**) absence and (**ii**,**iii**) presence of isovaleric acid. (**b**) *M. luteus* in the (**i**) absence and (**ii**,**iii**) presence of isovaleric acid. Cell surface blebbing is visible, with an altered shape and evidence of autolysis. (**c**) Cryo-SEM of *M. luteus* in the absence (**i**) and presence of isovaleric acid: (**ii**) edge of the zone of inhibition showing healthy cells alongside lysed cells, (**iii**) zone of inhibition showing islands of intact and unhealthy cells, (**iv**) details of healthy and dying cells, (**v**) micro-colony of healthy cells in the zone of inhibition and (**vi**) micro-colony of dying cells.

**Figure 7:**
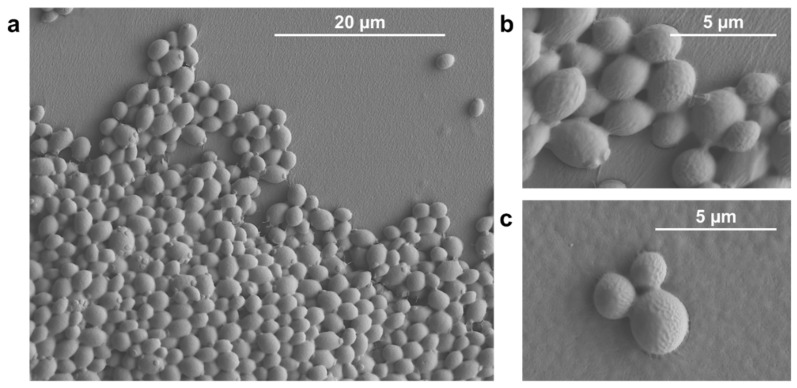
Cryo-scanning electron micrographs of *Candida glabrata* in the (**a**,**b**) absence (**c**) and presence of isovaleric acid.

The pattern of regrowth in filamentous fungi (*F. graminearum*) differed markedly, with growth being stunted partially or totally depending on the VOC (Figure 5A,B). Closer examination of the bacterial and yeast cells using conventional SEM and Cryo-SEM showed that cells had lysed (Figure 6 and Figure 7). Lysis was often preceded by unusual deposits appearing on the bacterial surface, followed by loss of form, then complete disappearance of the bacteria, leaving behind a “debris-free” clear zone (Figure 6). This pattern appeared to be true for both rod and spherical bacteria (Figure 6A,B). In some instances, micro-colonies and remnants of an extracellular matrix were observed at the interface of the surviving culture and clear zone (Figure 6A–C). The micro-colonies were made of several layers, with the surface cells clearly losing form when exposed to inhibitory VOCs (Figure 6C). No gross morphological changes were observed in yeast exposed to inhibitory VOCs, but cells had clearly disappeared in the clear (inhibition) zone, with the absence of any wall residue suggesting total digestion of the cell (Figure 7).

## 4. Discussion

This study showed that *M. brunneum* strains produced a wide array of VOCs with the profile (type and quantity of chemical) being influenced by the development stage of the fungus and cultural conditions. The EPF VOC profiles appear to vary with EPF species, isolate and culture conditions [22,25,30]. The unique blend of volatiles emitted by EPF is of significance from both ecological and biocontrol perspectives. It could explain why some EPF strains influence the behaviour of some invertebrates, while others do not [24,26]. Compounds that attract invertebrates could help with spore dispersal or form part of the pathogens’ “lure and kill” strategy [24,26]. Repellent compounds could reduce EPF efficacy. The implications for biocontrol are profound, as illustrated by studies conducted on sweet potato weevil and termites; these insects avoid virulent strains of *M. anisopliae* with the response being linked to their VOCs [23,30,38].

A novel and salient feature of this study was that *M. brunneum* produced VOCs with antimicrobial properties. The most potent antimicrobials were isovaleric acid and 1-octen-3-ol, which inhibited or killed bacteria, yeasts, filamentous fungi and the oomycete *P. ultimum*. In contrast, isoamyl formate, 3-octenone, cedrene and farnesene, although potent, were more restricted in their specificity. Some microbes were clearly more sensitive to *M. brunneum* VOCs than others. In order of sensitivity, *F. graminearum* was inhibited by far more compounds than any other test microbe, followed by *B. cinerea*, then *P. ultimum*. Of the test bacteria, *B. megaterium* was the most sensitive, followed by *B. thuringiensis*. This differential sensitivity may reflect some evolutionary arms race between EPF and competing microbes, especially plant pathogens, occupying the same niche. Since EPF, such as *M. brunneum*, are highly successful at colonizing plant roots and existing as endophytes, it is tempting to speculate that the capacity to produce antifungal VOCs evolved to enable the fungus to inhibit/eliminate potential competitors or fungi that could harm the host plant. EPF were shown to be antagonistic to plant pathogenic fungi via the production of antifungal metabolites and induction of the host’s defences [39]. However, the results of the current study suggested that *M. brunneum* VOCs could play a role in plant disease suppression and concomitantly contribute to healthy plant growth. 

Many non-EPF, such as *Muscodor heveae*, *Xylaria* sp., *Daldinia cf concentrica* and *Saccharomyces cerevisiae*, also produce antifungal VOCs that inhibit the growth of a wide range of phytopathogens, including *Sclerotinia sclerotiorum*, *Colletotrichum gloeosporioides*, *Botrytis cinerea* and *Phyllosticta citricarpa* [40,41,42,43,44,45,46]. Interestingly, one of the most inhibitory compounds produced by these fungi was methyl-1-butanol (isoamyl alcohol), which, in the current study, showed moderate inhibition of *F. graminearum* and *B. cinerea* and weak inhibition of *P. ultimum*. Some antifungal VOCs appear to be unique to specific fungi. For example, 6-pentyl-α-pyrone, trimethylamine and 3-methyl-acetate appear to be peculiar to *Trichoderma* species, *Geotrichum candidum* and *Muscodor albus*, respectively [47,48,49]. We identified several new antifungal VOCs, with the most potent being isovaleric acid, which also had strong antibacterial activity. Two compounds *M. brunneum* has in common with other fungi are 1-octen-3-ol and 3-octanone [50], both of which have strong antifungal activity, with the former responsible for self-inhibition of conidia in several fungi [51,52]. The current study showed that 1-octen-3-ol also had strong antibacterial properties. 

We are not aware of any study that explained the mechanism of inhibition. The current study showed that *Candida* yeasts and test bacteria exhibit similar patterns of sensitivity to *M. brunneum* VOCs, with the cells undergoing autolysis, ultimately leading to total cell degradation since no cell wall fragments or any other cell debris was evident in the inhibition zone. Biotic and environmental stresses are known to trigger autolysis in bacteria [53]. The degree of inhibition or lysis is clearly dependent upon the VOC dose but there is some evidence that the VOC must make direct contact with the target since cells hidden by other cells often survived and even flourished, presumably utilising the nutrients released from the adjacent lysed cells. In the soil environment, the lysate may be utilised by other microbes. Thus, EPF VOCs may influence the microbial profile in the rhizosphere. Indeed, analysis of the plant microbiome after *Metarhizium* amendment reveals an increase in the abundance of plant-growth-promoting organisms [54]. 

Only a tiny fraction of compounds produced by *M. brunneum* were screened; it is possible that the blend produced in nature is more potent than the individual compounds. In other words, some combination of VOCs may act synergistically, as reported for several bacteria species [19,55]. Synergies would conserve energy since fewer bioactive compounds need to be produced. Since *M. brunneum* produces a wide range of VOCs, the synergies between different combinations of VOCs may allow the fungus to compete with a wide range of microbial antagonists.

This study showed that cultural conditions can influence VOC profiles. This plasticity would allow *M. brunneum* to adapt to its environment and compete with indigenous microbes. Indeed, it would also explain the success of this and other EPF as they recovered from disparate habitats (soils, crops, etc.) around the world. This confirmed that these organisms possess the appropriate armoury to survive in what is recognised as a highly competitive environment [2,56]. There is much evidence showing that the microbial VOC profile is altered depending on environmental conditions, with novel compounds often being produced, especially under conditions of stress [19]. Stress factors may be environmental or biotic, such as exposure to antagonists, as illustrated by the mycophagous bacterium, i.e., *Collimonas fungivorans*, which only produces VOCs with strong antifungal activity in the presence of susceptible fungi [12]. 

The natural antimicrobial VOCs identified in this study have the potential for use as plant protection products. Their ephemeral nature and action through fumigation mean that they could be used to clean soils of harmful diseases or protect produce from post-harvest diseases. They would be relatively safer than many current chemical fumigants, which are highly toxic and often require long safety period. Indeed, many conventional chemical pesticides have been withdrawn due to their persistence and environmental pollution. 

## Data Availability

Data sharing not applicable.

## Supplementary Materials

The following supporting information can be downloaded at: https://www.mdpi.com/article/10.3390/jof8040326/s1, Table S1: Metarhizium brunneum volatile organic compounds (VOCs) tested for biocidal activity; Table S2: Bacterial, fungal and oomycete targets used to test VOC potency; Table S3: Metarhizium brunneum VOCs identified by NIST. The list shows compounds produced independent of strain, developmental stage (mycelium vs conidia), production in vivo or in vitro. The list includes compounds produced consistently as well as those produced intermittantly; Table S4: VOCs produced by Metarhizium brunneum ARSEF4556, ARSEF3297 and V275 on different substrates.
