# Supplementary material for: Antimicrobial Volatiles of the Insect Pathogen Metarhizium brunneum
Source: J Fungi (Basel). 2022 Mar 22;8(4):326. doi: 10.3390/jof8040326 (PMC9025432; doi:10.3390/jof8040326)
Supplement: Supplementary file 1 [file jof-08-00326-s001.zip › jof-1615104-supplementary.pdf]

# Antimicrobial volatiles of the insect-pathogen *Metarhizium brunneum*

Hummadi, E. H., Cetin, Y., Demirbek, M., Kardar, N.M., Khan, S., Coates, C.J., Eastwood, D.C., Dudley, E., Maffeis, T., Loveridge, E.J. & Butt, T.M.

**Table S1.** *Metarhizium brunneum* volatile organic compounds (VOCs) tested for biocidal activity.

| VOC (purity) | Synonyms | CAS Number | MW (g/mol) | Molecular formula |
|---|---|---|---|---|
| Isoamyl alcohol (99%) | 3-Methyl-1-butanol, Isopentyl alcohol | 123-51-3 | 88.15 | $C_5H_{12}O$ |
| Isoamyl formate (95%) | Isopentyl formate, 3-Methylbutyl formate, Isoamyl methanoate, I sopentyl methanoate, formic acid isoamyl ester | 110-45-2 | 116.16 | $C_6H_{12}O_2$ |
| Methyl isovalerate (98%) | Methyl Isopentanoate, Methyl 3-methylbutanoate, Isovaleric acid methyl ester | 556-24-1 | 116.16 | $C_6H_{12}O_2$ |
| 3-Octanone (98%) | Ethyl amyl ketone, Ethyl pentyl ketone | 106-68-3 | 128.21 | $C_8H_{16}O$ |
| (R)-(+)-Limonene (97%) | (+)-p-Mentha-1,8-diene, (+)-Carvene, (R)-4-Isopropenyl-1-methyl-1-cyclohexene | 5989-27-5 | 136.23 | $C_{10}H_{16}$ |
| Isovaleric acid (99%) | 3-Methylbutanoic acid, 3-Methylbutyric acid | 503-74-2 | 102.13 | $C_5H_{10}O_2$ |
| 1-Octen-3-ol (98%) | Pentyl vinyl carbinol | 3391-86-4 | 128.21 | $C_8H_{16}O$ |
| Farnesene (mixture of isomers) | α-Farnesene, 3,7,11-trimethyl-1,3,6,10-dodecatetraene | 502-61-4 | 204.35 | $C_{15}H_{24}$ |
| (−)-α-Cedrene (95%) | (1S,2R,5S)-2,6,6,8-Tetramethyltricyclo[5.3.1.01.5]undec-8-ene | 469-61-4 | 204.35 | $C_{15}H_{24}$ |
| 2,3-Butanediol (98%) | 2,3-Butylene glycol | 513-85-9 | 90.12 | $C_4H_{10}O_2$ |
| 1-Octene (98%) | Octene-1, Octylene; n-octene, Hexylethylene, Oct-1-ene | 111-66-0 | 112.21 | $C_8H_{16}$ |
| Undecane (99%) | n-Undecane, Hendecane | 1120-21-4 | 156.31 | $C_{11}H_{24}$ |
| Tridecane (99%) | n-Tridecane | 629-50-5 | 184.36 | $C_{13}H_{28}$ |
| Damascenone (98%) | β-Damascenone, 1-(2,6,6-Trimethylcyclohexa-1,3-dien-1-yl)-2-buten-1-one | 23696-85-7 | 190.28 | $C_{13}H_{18}O$ |

**Table S2.** Bacterial, fungal and oomycete targets used to test VOC potency.

| Microbe | Group | Ecological significance | Source |
|---|---|---|---|
| *Micrococcus luteus,* NCIB 8553 | Gram + | Widespread (soil, water, skin flora) | Llinos Harris, Swansea University |
| *Escherichia coli,* NCIB 8277 | Gram – | Human Intestine | Llinos Harris, Swansea University |
| *Pantoea agglomerans,* NCTC 9381 | Gram – | Widespread (soil, plants, insect gut), plant pathogen (fire blight) | Llinos Harris, Swansea University |
| *Bacillus megaterium,* NCIB 10342 | Gram + | Soil bacterium, BCA use to control plant diseases and plant parasitic nematodes. | Llinos Harris, Swansea University |
| *Bacillus subtilis,* NCIB 3610 | Gram + | Soil bacterium, commensal in human gut | Llinos Harris, Swansea University |
| *B. thuringiensis* subsp. *Galleria* (H-serotype V) strain 69-6 | Gram + | Soil bacterium, BCA used to control insect pests | ISEA bacterial collection |
| *Pseudomonas aeruginosa,* PA01 | Gram – | Widespread (soil, water, skin flora) | Angus Buckling, Oxford University |
| *Staphylococcus aureus,* ATCC 6538P | Gram + | Human and animal surfaces | American Type Culture Collection |
| *Candida albicans,* NCYC 3778 | Yeast | Human pathogen | National Collection of Yeast Cultures |
| *Candida glabrata,* NCYC 3537 | Yeast | Human pathogen | National Collection of Yeast Cultures |
| *Pythium ultimum* | Oomycete | Plant pathogen (Damping off) | Jane Nicklin, University of London |
| *Botrytis cinerea* | Fungus | Plant pathogen (Gray mould) | Naresh Magan, Cranfield University |
| *Fusarium graminearum* | Fungus | Plant pathogen (Cereal head blight) | Naresh Magan, Cranfield University |

**Table S3.** *Metarhizium brunneum* VOCs identified by NIST . The list shows compounds produced independent of strain, developmental stage (mycelium vs conidia), production *in vivo* or *in vitro*. The list includes compounds produced consistently as well as those produced intermittantly.

| Compound | MW (g/mol) | Compound | MW (g/mol) |
|---|---|---|---|
| **Alkanes** | | **Esters** | |
| Pentane | 72.15 | Ethyl acetate | 88.10 |
| Heptane | 100.21 | Isoamyl formate | 116.15 |
| Octane | 114.23 | Methyl isovalerate | 116.15 |
| 1-Methyl-2-pentylcyclohexane | 168.32 | Isoamyl acetate | 130.18 |
| Dodecane | 170.34 | Methyl (3-hydroxycyclopentyl) acetate | 158 |
| 3-Methyltridecane | 198.39 | Methyl 2-ethylhexanoate | 172.26 |
| 5-Methyltridecane | 198.38 | **Monoterpenes** | |
| Tetradecane | 198.39 | Limonene | 136.24 |
| 7-Methylpentadecane | 226.44 | Limonene-6-ol pivalate | 236.35 |
| Hexadecane | 226.44 | **Sesquiterpenes** | |
| Eicosane | 282.55 | Acoradiene | 204.35 |
| 3-Methyleicosane | 296.57 | Bergamotene | 204.35 |
| **Alkenes** | | Bisabolene | 204.35 |
| 1-Octene | 112.24 | Cardinene | 204.35 |
| 1,3-Octadiene | 110.2 | Cedrene | 204.35 |
| 2-ethenyl-1,3,3-trimethylcyclohexene | 150.26 | Chamigene | 204.35 |
| 1-Undecene | 154.29 | Copaene | 204.35 |
| 4,4-dimethyl-2-neopentyl-1-pentene | 168.31 | Cubebene | 204.35 |
| **Alcohols** | | Di-epi-α-cedrene | 204.35 |
| Isoamyl alcohol | 88.14 | Elemene | 204.35 |
| 2,3-butanediol | 90.12 | Farnesene | 204.36 |
| 2-Phenylethanol | 122.16 | Himachala-2,4-diene | 204.35 |
| 1-Octen-3-ol | 128.22 | Himachalene | 204.35 |
| **Ketones** | | Isocaryophyllene | 204.35 |
| 3-Octanone | 128.21 | Isoledene | 204.35 |
| 5,9-dimethyl-5,8-decadiene-2-one | 180 | Patchoulene | 204.35 |
| **Carboxylic acids** | | β-sesquiphellandrene | 204.35 |
| Acetic acid | 605 | Thujopsene | 204.35 |
| Isovaleric acid | 102.13 | Valencene | 204.35 |
| 2-Methylhexanoic acid | 130.18 | α-seinene | 204.35 |
| **Other compounds** | | α-zingiberene | 204.35 |
| 3-Hydroxy-2-butanone | 88.10 | Cubenol | 222.37 |
| Methoxybenzene | 108.14 | α-bisaboial | 222.37 |
| Dimethoxybenzene | 138.17 | Damascenone | 190.29 |

**Table S4.** VOCs produced by *Metarhizium brunneum* ARSEF4556, ARSEF3297 and V275 on different substrates. The percentage values are proportional to the compound with the highest peak (100%) in the chromatogram. (Values are means of 5 replicates). Abbreviations: Rt = Retention time, MW = Molecular weight, OSM = Osmotic Stress Medium, HCN = High C:N medium, ICN = Intermediate C:N medium, LCN = Low C:N medium, – = Not observed in this medium, * = Authentic compounds having the same RT and MS as synthetic standard, Tr. = Trace amount.

| Compound | $R_t$ (min) | MW | ARSEF 4556 (After 7 days – no conidia) | | | | ARSEF 4556 (After 14 days – conidia) | | | | V275 (After 7 days) | | | | V275 (After 14 days) | | | | ARSEF 3297 (After 7 days) | | | | ARSEF 3297 (After 14 days) | | | |
|---|---|---|---|---|---|---|---|---|---|---|---|---|---|---|---|---|---|---|---|---|---|---|---|---|---|---|
| | | | OSM | HCN | ICN | LCN | OSM | HCN | ICN | LCN | OSM | HCN | ICN | LCN | OSM | HCN | ICN | LCN | OSM | HCN | ICN | LCN | OSM | HCN | ICN | LCN |
| Pentane* | | 72 | - | - | - | - | 65.15 | - | - | - | - | - | - | - | - | - | - | - | - | - | - | - | - | - | - | - |
| Ethyl acetate* | | 88 | - | - | - | - | - | - | - | - | - | 43.04 | 37.95 | 16.42 | - | - | - | - | - | - | - | - | - | - | - | - |
| Acetic acid* | | 60 | - | 100 | 100 | 100 | - | - | - | - | 100 | 85.76 | - | - | - | - | - | - | 100 | 100 | 100 | 100 | - | - | - | - |
| Heptane* | 1.534 | 100 | - | - | - | - | 22.60 | - | - | - | - | - | - | - | - | - | - | - | - | - | - | - | - | - | - | - |
| 3-hydroxy-2-butanone* (Acetoin) | 2.015 | 88 | - | - | - | - | - | - | - | - | - | 49.38 | 33.58 | 32.25 | - | 57.9 | 73.73 | 100 | - | - | - | - | - | - | - | - |
| Isoamyl alcohol* | 2.075 | 88 | 100 | - | - | - | 61.85 | - | - | - | 2.30 | 20.44 | 100 | 100 | - | - | - | - | 34.29 | - | - | - | - | - | - | - |
| Isoamyl formate* | 2.770 | 116 | - | - | - | - | - | - | - | - | - | - | 4.88 | - | - | - | - | - | - | - | - | - | - | - | - | - |
| Methyl isovalerate* | 2.945 | 116 | - | - | - | - | - | - | - | - | - | - | 2.01 | - | - | - | - | - | - | - | - | 6.73 | - | - | - | - |
| 1-Octene* | 3.520 | 112 | - | - | - | - | - | 0.86 | 1.62 | - | - | - | - | - | 11.13 | 2.67 | 102 | 3.89 | - | - | - | - | 8.27 | - | 6.62 | - |
| Octane* | 4.123 | 114 | - | - | - | - | 10.70 | 0.57 | 1.12 | 25.6 | - | - | - | - | - | - | - | - | - | - | - | - | - | - | - | - |
| 2,3-butanediol* (R or S isomer) | 4.168 | 90 | - | - | - | - | - | - | - | - | - | Tr. | Tr. | - | - | 43.43 | 44.49 | 4.16 | - | - | - | - | - | - | - | - |
| 2,3-butanediol* (R or S isomer) | 4.479 | 90 | - | - | - | - | - | - | - | - | - | 100 | 65.96 | - | - | 100 | 100 | 19.48 | - | - | - | - | - | - | - | - |
| 1,3-Octadiene | 4.662 | 110 | - | - | - | - | - | 18.39 | 36.40 | 100 | - | - | - | - | 56.20 | 14.92 | 88.05 | 34.14 | - | - | - | - | 100 | - | 100 | - |
| Isovaleric acid* | 4.667 | 102 | 7.79 | 9.84 | 11.82 | 8.78 | - | - | - | - | 8.46 | 20.40 | - | - | - | - | - | - | 8.91 | 4.79 | 4.18 | 12.97 | - | - | - | - |
| Isopentyl acetate* | 4.708 | 130 | - | - | - | - | - | - | - | - | - | 105 | 9.40 | 7.5 | - | - | - | - | - | - | - | - | - | - | - | - |
| 2-Methylhexanoic acid | 5.462 | 116 | - | - | - | - | - | - | - | - | - | - | - | - | - | - | - | - | 4.04 | 2.31 | - | 3.76 | - | - | - | - |
| 1-Octen-3-ol* (R, S isomer) | 7.099 | 128 | - | - | - | - | - | - | - | - | - | - | - | - | 100 | 12.39 | 32.89 | 18.03 | - | - | - | - | - | - | - | - |
| 3-Octanone* | 7.101 | 128 | - | - | - | - | - | - | - | - | - | - | - | - | - | - | - | 20.53 | - | - | - | - | - | - | - | - |

Supplementary information

| | | | | | | | | | | | | | | | | | | | | | | | | | | |
|---|---|---|---|---|---|---|---|---|---|---|---|---|---|---|---|---|---|---|---|---|---|---|---|---|---|---|
| 4,4-Dimethyl-2-neopentyl-1-pentene | 7.353 | 168 | - | - | - | - | - | - | - | - | - | - | - | - | - | - | - | - | - | - | - | - | - | 47.57 | 30.28 | 100 |
| 1-Methyl-2-pentyl cyclohexane | 11.341 | 168 | - | - | - | - | - | 10.16 | - | 6.115 | - | - | - | - | - | - | - | - | - | - | - | - | - | 43.93 | 22.40 | 38.47 |
| Methyl 2-ethylhexanoate* | 11.636 | 158 | 19.20 | 1.92 | 7.52 | 3.36 | 3.83 | - | 1.68 | - | 8.40 | 11.1 | 12.93 | 29.68 | - | 1.79 | 1.40 | 5.85 | 3.81 | 1.73 | 3.28 | 20.56 | - | - | - | 31.62 |
| Methyl 3-hydroxy cyclopentyl acetate | 11.869 | 158 | 14.34 | - | - | - | 3.14 | - | - | - | - | - | - | 5.87 | - | - | - | - | - | - | - | - | - | - | - | - |
| 1-Undecene | 14.090 | 154 | - | - | - | - | - | - | - | - | - | - | 9.28 | 56.61 | - | - | 3.66 | 19.25 | - | - | - | - | - | - | - | - |
| 2-Phenylethanol* | 15.780 | 122 | - | - | - | - | 18.54 | - | - | - | - | - | 6.52 | - | - | - | - | - | - | - | - | - | - | - | - | - |
| Cedrene* | 16.454 | 204 | - | - | - | - | 23.37 | Tr. | 46.60 | 22.81 | 14.2 | Tr. | - | 4.85 | 17.64 | 9.30 | 5.53 | 13.20 | 9.06 | 1.72 | 4.0 | 4.19 | 6.89 | 100 | 14.10 | 58.5 |
| Bergamotene* | 17.883 | 204 | - | - | - | 1.42 | 8.77 | 12.41 | 5.50 | Tr. | - | - | - | - | - | - | - | - | - | - | - | - | - | - | - | - |
| Farnesene* | 30.641 | 204 | 24.05 | 1.81 | 5.83 | 38.76 | 100 | 100 | 100 | 14.05 | - | - | - | - | - | - | - | - | - | - | - | - | - | - | - | 54.3 |
